# Impact of hyperuricemia on mortality related to aortic diseases: a 3.8-year nationwide community-based cohort study

**DOI:** 10.1038/s41598-020-71301-6

**Published:** 2020-08-31

**Authors:** Yoichiro Otaki, Tetsu Watanabe, Tsuneo Konta, Masafumi Watanabe, Koichi Asahi, Kunihiro Yamagata, Shouichi Fujimoto, Kazuhiko Tsuruya, Ichiei Narita, Masato Kasahara, Yugo Shibagaki, Kunitoshi Iseki, Toshiki Moriyama, Masahide Kondo, Tsuyoshi Watanabe

**Affiliations:** 1 Department of Cardiology, Pulmonology, and Nephrology, Yamagata University School of Medicine, 2-2-2 Iida-Nishi, Yamagata, 990-9585 Japan; 2 Steering Committee of Research on Design of the Comprehensive Health Care System for Chronic Kidney Disease (CKD) Based on the Individual Risk Assessment by Specific Health Check, Fukushima, Japan

**Keywords:** Cardiology, Endocrinology, Risk factors

## Abstract

Despite advances in medicine, aortic diseases (ADs) such as aortic dissection and aortic aneurysm rupture remain fatal with extremely high mortality rates. Owing to the relatively low prevalence of AD, the risk of AD-related death has not yet been elucidated. The aim of the present study was to examine whether hyperuricemia is a risk factor for AD-related mortality in the general population. We used a nationwide database of 474,725 subjects (age 40–75 years) who participated in the annual “Specific Health Check and Guidance in Japan” between 2008 and 2013. There were 115 deaths from aortic dissection and aortic aneurysm rupture during the follow-up period of 1,803,955 person-years. Kaplan–Meier analysis revealed that subjects with hyperuricemia had a higher rate of AD-related death than those without hyperuricemia. Multivariate Cox proportional hazard regression analysis demonstrated that hyperuricemia was an independent risk factor for AD-related death in the general population. The net reclassification index was improved by addition of hyperuricemia to the baseline model. This is the first report to demonstrate that hyperuricemia is a risk factor for AD-related death, indicating that hyperuricemia could be a crucial risk for AD-related death in the general population.

## Introduction

Owing to the westernization of eating habits, hyperuricemia is becoming an increasing public health problem associated with gout arthritis as well as cardiovascular risk and mortality^1^. Hyperuricemia was previously considered a mere bystander condition to cardiovascular diseases, as it is closely associated with cardiovascular risk factors such as hypertension (HT), diabetes mellitus (DM), chronic kidney disease (CKD), and metabolic syndrome^2–5^. Recent reports revealed a relationship between hyperuricemia and stroke, cardiovascular disease, and deaths^6–8^. On the other hand, although patients with aortic diseases (ADs), such as aortic dissection and aortic aneurysm rupture, reportedly had higher levels of uric acid than those without ADs^9–11^, the impact of hyperuricemia on AD-related mortality in the general population remains undetermined.

AD is a devastating clinical problem that can cause sudden death^12–14^. Notably, the prevalence of aortic dissection is still increasing in developing countries^15^. Despite advances in medicine, it remains difficult to save patient lives after the onset of AD, and almost all patients with AD die before hospital arrival. Furthermore, the mortality rate of AD at 1 month after symptom onset reaches approximately 50% despite treatment^16,17^. Therefore, it is crucial to identify high-risk persons and prevent the development of AD in the general population through health check-ups. Because the prevalence of AD is relatively low, there has been no prospective cohort study with sufficient data to analyze whether hyperuricemia could be a risk factor for AD in the general population until now.

The present study aimed to examine whether hyperuricemia is a pivotal risk factor for AD-related death in the general population.

## Results

### Baseline characteristics and comparison of clinical characteristics between subjects with and those without hyperuricemia

The baseline characteristics of the 203,087 men and 271,638 women are shown in Table 1. HT, DL, and DM were identified in 279,480 (59%), 234,204 (49%), and 44,592 (9.4%) of the subjects, respectively. The mean serum uric acid level was 5.3 mg/dL. Hyperuricemia was identified in 51,157 (11%) of all subjects, including 44,467 (22%) men and 6,690 (2%) women.

**Table1:** Comparison of clinical characteristics between patients with and without hyperuricemia.

Variables	All subjects n = 474,725	Hyperuricemia (−) n = 423,568	Hyperuricemia (+) n = 51,157	P value
Age	62.8 ± 8.8	62.9 ± 8.8	61.5 ± 9.4	< 0.0001
Male, n (%)	203,087 (43%)	158,620 (37%)	44,467 (87%)	< 0.0001
BMI, kg/m^2^	23.5 ± 3.4	23.3 ± 3.4	25.1 ± 3.5	< 0.0001
Hypertension, n (%)	279,480 (59%)	242,236 (57%)	37,244 (73%)	< 0.0001
Dyslipidemia, n (%)	234,204 (49%)	202,347 (48%)	31,857 (62%)	< 0.0001
Diabetes mellitus, n (%)	44,592 (9.4%)	39,296 (9.3%)	5,296 (10.4%)	< 0.0001
Smoking, n (%)	72,586 (15%)	59,474 (14%)	13,112 (26%)	< 0.0001
Previous cardiovascular disease, n (%)	16,705 (3.5%)	14,262 (3.4%)	2,443(4.8%)	< 0.0001
Previous cerebrovascular disease, n (%)	25,646 (5.4%)	22,102 (5.2%)	3,544 (6.9%)	< 0.0001
**Biochemical data**				
Uric acid (mg/dL)	5.3 ± 1.0	5.0 ± 1.1	7.9 ± 0.8	< 0.0001
eGFR (ml/min/1.73 m^2^)	75.6 ± 15.4	79.5 ± 15.4	58.3 ± 15.3	< 0.0001
HbA1c (%)	5.38 ± 0.74	5.38 ± 0.75	5.36 ± 0.64	0.0001
Fasting blood glucose (mg/dL)	98 ± 22	98 ± 22	102 ± 20	< 0.0001
**Medications**				
Anti-hypertensive drug, n (%)	141,321 (30%)	121,392 (29%)	19,929 (39%)	< 0.0001
Anti-diabetic drug, n (%)	26,206 (5.5%)	23,330 (5.5%)	2,876 (5.6%)	0.2876
Anti-dyslipidemia drug, n (%)	69,607 (14.7%)	63,581 (15.0%)	6,026 (11.8%)	< 0.0001

Data are expressed as mean ± SD, number (percentage), or median (interquartile range).

*BMI* body mass index, *eGFR* estimated glomerular filtration rate, *HbA1c* glycosylated hemoglobin A1c.

Subjects with hyperuricemia were younger and more likely to be men; have HT, DL, DM, previous cardiovascular disease, or previous cerebrovascular disease; be a current smoker; or be taking anti-hypertensive and anti-DL drugs than those without hyperuricemia. Further, subjects with hyperuricemia showed higher FBG and HbA1c levels and lower eGFR than those without hyperuricemia (Table 1).

### Hyperuricemia and AD-related deaths

**Figure 1:**
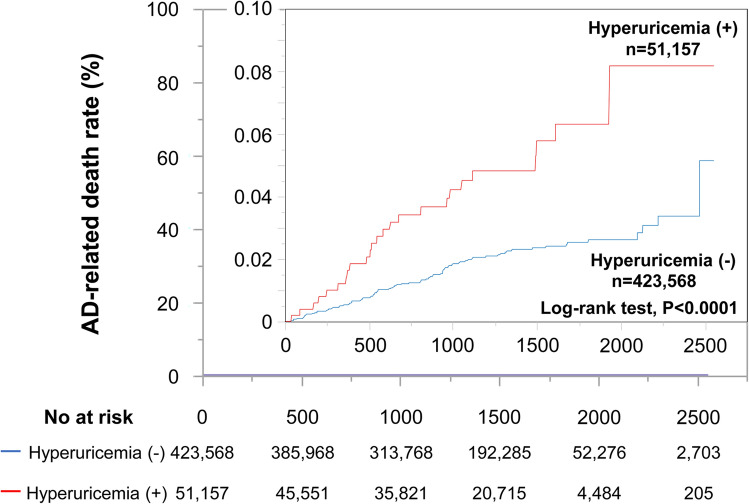
Kaplan–Meier analysis of aortic artery disease (AD)-related deaths in subjects with versus those without hyperuricemia.

All subjects were prospectively followed up for 1,803,955 person-years (median follow-up period, 3.8 years). During the follow-up period, there were 115 AD-related deaths. Kaplan–Meier analysis demonstrated that subjects with hyperuricemia had a higher rate of AD-related death than those without hyperuricemia (Fig. 1).

**Figure 2:**
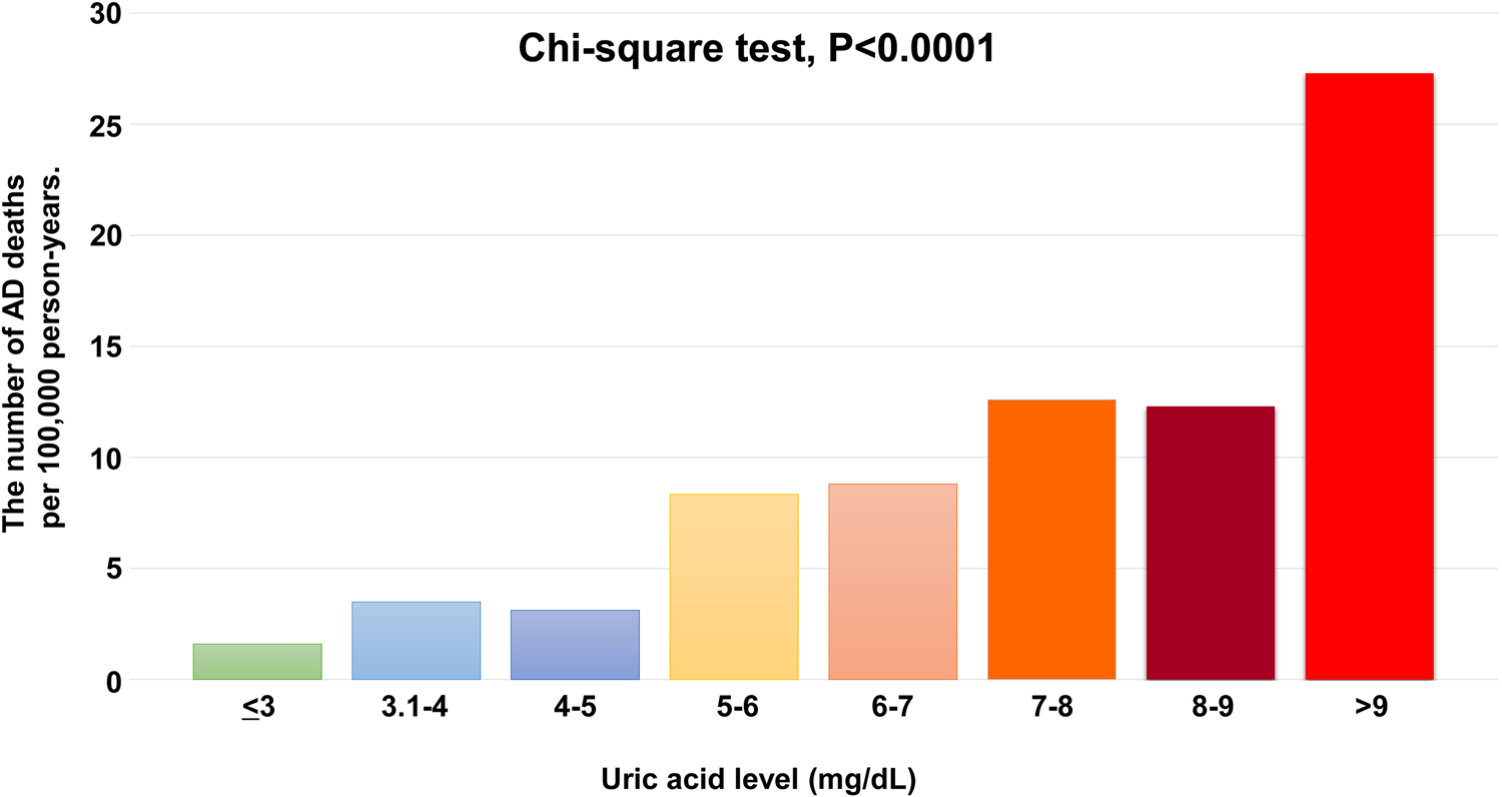
Association of aortic disease (AD)-related death per 100,000 person-years with serum uric acid levels.

To examine whether a J-curve association between uric acid and AD-related deaths existed in the general population, we divided all subjects into 8 groups based on serum uric acid level: uric acid ≤ 3 mg/dL group (n = 16,138; 62,615 person-years), uric acid = 3.1–4 mg/dL group (n = 73,470; 285,064 person-years), uric acid = 4.1–5 mg/dL group (n = 133,937; 511,639 person-years), uric acid = 5.1–6 mg/dL group (n = 122,198; 455,798 person-years), uric acid = 6.1–7 mg/dL group (n = 77,825; 284,061 person-years), uric acid = 7.1–8 mg/dL group (n = 35,386; 127,389 person-years), uric acid = 8.1–9 mg/dL group (n = 11,532; 40,708 person-years), and uric acid > 9 mg/dL group (n = 4,239; 14,666 person-years). Incident AD-related deaths increased with increasing uric acid level (Fig. 2), indicating no obvious J-curve association between uric acid level and AD-related deaths in the general population.

To determine the risk factors for predicting AD-related deaths, we performed univariate and multivariate Cox proportional hazard regression analyses. In the univariate analysis, uric acid was significantly associated with AD-related mortality (Table 2), while age, sex, HT, smoking, previous cardiovascular disease, previous cerebrovascular disease, and eGFR were also related to AD-related mortality. Multivariate Cox proportional hazard regression analysis demonstrated that hyperuricemia was an independent predictor of future AD-related deaths after the adjustment for age, sex, HT, smoking, previous cardiovascular disease, previous cerebrovascular disease, and eGFR (hazard ratio 1.166; 95% confidence interval 1.012–1.342; P = 0.0340; Table 2).

**Table 2:** Univariate and multivariate Cox proportional hazard analyses of predicting AD-related death.

Variables	Hazard ratio	95% confidence interval	P value
**Univariate analysis**			
Age, per-1 year increase	1.082	1.049–1.164	< 0.0001
Sex	2.306	1.588–3.388	< 0.0001
Hypertension	5.704	3.328–10.660	< 0.0001
Smoking	1.917	1.237–2.885	0.0043
Previous cardiovascular disease	2.553	1.397–4.312	0.0035
Previous cerebrovascular disease	2.486	1.168–4.637	0.0205
eGFR, per-1SD increase	0.566	0.462–0.694	< 0.0001
Uric acid, per-1SD increase	1.359	1.227–1.505	< 0.0001
**Multivariate analysis**			
Age, per-1 year increase	1.064	1.029–1.100	0.0002
Sex	1.393	0.899–2.108	0.1386
Hypertension	4.206	2.387–8.126	< 0.0001
Smoking	2.208	1.406–3.472	0.0006
Previous cardiovascular disease	1.588	0.860–2.714	0.1323
Previous cerebrovascular disease	1.408	0.656–2.660	0.3537
eGFR, per-1SD increase	0.710	0.574–0.879	0.0017
Uric acid, per-1SD increase	1.166	1.012–1.342	0.0340

*AD* aortic artery disease, *eGFR* estimated glomerular filtration rate.

### Improvement of reclassification by the addition of hyperuricemia to predict AD-related mortality

To examine whether the model fit and discrimination improve with the addition of hyperuricemia to the basic predictors such as age, sex, HT, smoking, previous cardiovascular disease, previous cerebrovascular disease, and eGFR, we evaluated the improvement of the C-index and NRI. Baseline model includes age, sex, HT, smoking, previous cardiovascular disease, previous cerebrovascular disease, and eGFR. The ROC curve analysis demonstrated no significant difference in the C-index between the baseline model and that with hyperuricemia. However, NRI was significantly improved by the addition of hyperuricemia to the baseline model (NRI 0.2092; 95% confidence interval 0.0266–0.3918; P = 0.0247; Table 3).

**Table 3:** Statistics for model fit and improvement with the addition of uric acid on the prediction of AD-related death.

	C index	NRI (95% CI, P value)
Baseline model	0.7551	Reference
+ Uric acid	0.7609 (P = 0.0937)	0.2092 (0.0266–0.3918, P = 0.0247)

Baseline model includes age, gender, HT, smoking, previous cardiovascular disease, previous cerebrovascular disease, and eGFR.

*eGFR* estimated glomerular filtration rate, *HT* hypertension, *NRI* net reclassification index, *95% CI* 95% confidence interval.

### Sex-based difference in the abnormal cutoff value of uric acid for AD-related death

**Figure 3:**
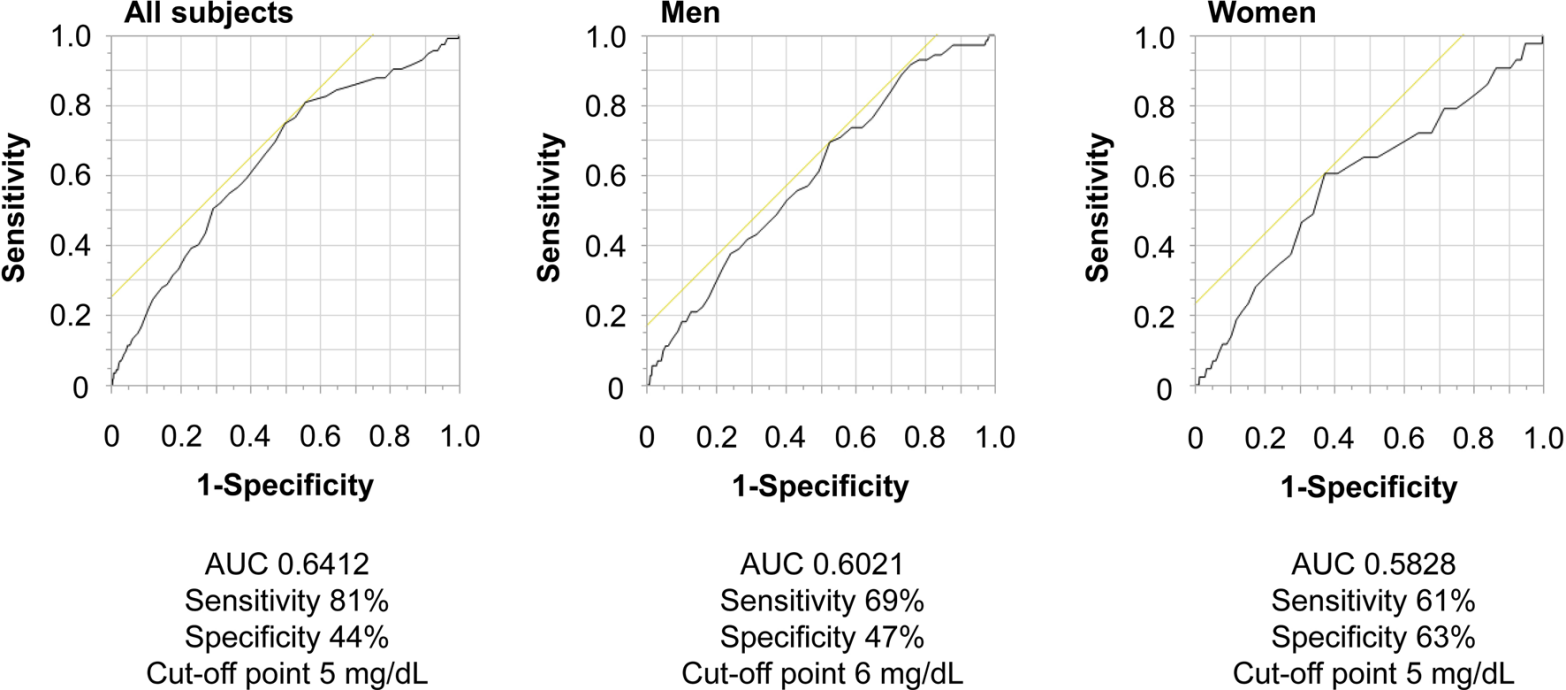
Receiver operating characteristic curve of aortic disease-related mortality in all subjects, in men, and in women. *AUC* area under the curve.

Finally, we constructed ROC curves for all subjects, for men, and for women and calculated the abnormal cutoff values for AD-related deaths in the general population. As shown in Fig. 3, the abnormal cutoff values for AD-related mortality differed by sex. The cutoff value for AD-related death in men and women were 6.0 and 5.0 mg/dL, respectively. Detailed results about sex difference in the impact of hyperuricemia on the AD-related death were provided in the Supplementary Information.

## Discussion

The main findings of the present study were as follows: (1) Kaplan–Meier analysis demonstrated that subjects with hyperuricemia had a higher rate of AD-related death; (2) incident AD-related death linearly increased with increasing uric acid level; (3) multivariate analysis demonstrated that hyperuricemia was an independent predictor of AD-related death; (4) the addition of hyperuricemia to cardiovascular risk factors improved the prediction of AD-related death in the general population; and (5) the abnormal cutoff values for AD-related mortality differed by sex in the general population.

The prevalence of hyperuricemia reached 20% in men and 5% in women in 2010, and a further increase is expected in Japan^1^. In the present study, the prevalence of hyperuricemia was 11% in apparently healthy subjects (22% in men, 2% in women). In accordance with previous reports, subjects with hyperuricemia in this study had a higher prevalence of HT, obesity, DM, CKD, and smoking than those without hyperuricemia. Therefore, whether hyperuricemia is associated with cardiovascular disease directly or indirectly remains controversial. The present study demonstrated that hyperuricemia was associated with AD-related mortality independent of confounding risk factors.

Several reports have indicated that serum uric acid level is associated with cardiovascular disease, stroke, and mortality in a J-curve manner^18–20^. This is explained by the fact that, because uric acid has antioxidant properties, hypouricemia could be a risk factor for cardiovascular disease and stroke similar to hyperuricemia. Different from these reports, our results showed that hypouricemia was not associated with AD-related death in the general population.

Although recent advances in clinical and basic AD research have uncovered the important role of the renin–angiotensin–aldosterone system in the development of AD^21–23^, the precise mechanism by which hyperuricemia causes AD is yet to be fully elucidated. Medial degeneration is a common histologic characteristic in AD and considered a risk factor for aortic aneurysm rupture and aortic dissection. Medial degeneration is reportedly related to aging and HT and is observed in patients with connective tissue disease^24–26^. Interestingly, subjects with hyperuricemia in this study were younger than those without hyperuricemia, and the significant association between hyperuricemia and AD-related mortality was maintained after the adjustment for age and HT. Therefore, other mechanisms should be examined. Xanthine oxidase-induced oxidative stress and urate deposition-induced inflammasome activation are the major causes of the development of hyperuricemia-related diseases such as gout, cardiovascular diseases, and CKD^27–30^. Because the abnormal cutoff value in men and women for AD-related death was < 7.0 mg/dL, which is an abnormal cutoff value for urate deposition, we speculated that xanthine oxidase-induced oxidative stress might be involved in the development of AD. Oxidative stress induces medial degeneration and is suggested to be a pathophysiology of AD development^31–33^. Oxidative stress in the aortic wall is derived from two major sources: the NADPH-dependent pathway and the xanthine oxidase pathway^34^. Uric acid is the final product of dietary and endogenous purines and is generated by xanthine oxidase^27,35^. Uric acid is reportedly found in the aortic vascular wall^34^. Esen et al. demonstrated that serum uric acid level is correlated with total antioxidant capacity and could be a marker of oxidative stress in patients with a dilated ascending aorta^10^. The xanthine oxidase–induced production of reactive oxygen species (ROS) such as hydrogen peroxide and superoxide is increased in proportion with the uric acid production in the aortic vascular wall^27^. An experimental study demonstrated that xanthine oxidase-induced ROS results in aortic wall oxidative stress and is inhibited by the xanthine oxidase inhibitor^35^. In addition to xanthine oxidase-induced ROS, hyperuricemia contributes to the development of atherosclerosis through endothelial dysfunction, platelet aggregation, and inflammation, leading to aortic wall weakness^36,37^. These findings supported our hypothesis that hyperuricemia is associated with the development of AD. Recently, Febuxostat for Cerebral and CaRdiorenovascular Events PrEvEntion StuDy (FREED) demonstrated that xanthine oxidase inhibitor reduced the risk for composite events including aortic aneurysm and dissection^38^. Further studies are needed to clarify whether treatment for hyperuricemia could prevent AD-related death or not.

Subjects with a serum uric acid level > 9 mg/dL had an undoubtedly high incidence of AD-related death, suggesting that anti-hyperuricemia drugs are recommended for these subjects as well as to prevent gout. Since this is a prospective observational study, we could not determine the target value of serum uric acid level to prevent AD-related death. However, the abnormal cutoff value of serum uric acid level for AD-related mortality in men was 6.0 mg/dL, which is equal to the treatment target value in patients with gout^39^. On the other hand, the abnormal cutoff value of serum uric acid level in women was 5.0 mg/dL. Although the definition of hyperuricemia was the same between men and women in Japan, several reports demonstrated a sex-based difference in the association between uric acid level and cardiovascular disease^40,41^. In light of these reports, we speculated that there might be a sex-based difference in the impact of uric acid on AD-related mortality (Supplementary Information).

The strengths of the present study include its large sample size, prospective follow-up design, and nationwide data source. Therefore, our results are well generalized and highly reliable. However, it also has some limitations. First, we assessed the serum uric acid level at only 1 time point. The medical treatment of hyperuricemia may affect AD-related mortality, although the effect of anti-hyperuricemia drugs on AD has never been examined. Second, we did not obtain data on AD development or therapies such as surgical and endovascular aortic repair. Although AD could be fatal, some subjects survived, probably owing to treatment. Thus, we underestimated the impact of hyperuricemia on the development of AD. Third, hyperuricemia is derived from the imbalance between uric acid production and excretion and classified into three types: underexcretion, extrarenal urate underexcretion, and urate overproduction^43^. We could not determine whether the impact of hyperuricemia on AD-related mortality differed among these types. Fourth, since we defined AD-related death from death certificate without validation of death certificate diagnosis for AD, we could not eliminate misclassification or underestimation of AD-related deaths. Fifth, serum uric acid level was reported to be affected by anti-hypertensive, anti-dyslipidemia, and anti-diabetic drugs^42–44^. Unfortunately, we have no data about details of medications. Thus, we could not eliminate the effect of medicine on the serum uric acid level. Finally, NRI was reported to be often biased by addition of unnecessary predictor and the use of risk models that do not fit the test data^45^. Since we included the established risk factors in the NRI analysis, the obtained result was reliable.

We demonstrated that hyperuricemia is a novel risk factor for AD-related deaths independent of risk factors. Notably, NRI was significantly improved by the addition of hyperuricemia, indicating that hyperuricemia could be added to the established risk factors. The main finding of the present study is that hyperuricemia is an independent predictor for AD-related death. In conclusion, hyperuricemia is a novel risk factor for AD-related deaths in the general population and could be a therapeutic target to prevent sudden death.

## Methods

### Study population

This study is part of the ongoing “Research on Design of the Comprehensive Health Care System for CKD Based on Individual Risk Assessments by Specific Health Check” for all inhabitants of Japan aged 40–74 years covered by the Japanese national health insurance. We used the data obtained from the following 16 prefectures (i.e., administrative regions): Hokkaido, Tochigi, Saitama, Chiba, Nagano, Niigata, Ishikawa, Fukui, Gifu, Hyogo, Tokushima, Fukuoka, Saga, Nagasaki, Kumamoto, and Okinawa. We collected data for 204,984 men and 273,561 women (total, 664,927) who participated in health check-ups between 2008 and 2013. The serum uric acid level was measured in 478,545 individuals; among them, 3,743 were excluded from this study owing to previous kidney dysfunction and 77 were excluded owing to missing essential data. Therefore, 203,087 men and 271,638 women were finally included in the study.

### Definition of cardiovascular risk

HT was defined as a systolic blood pressure ≥ 140 mmHg, diastolic blood pressure ≥ 90 mmHg, or the use of antihypertensive medication. DM was defined as a fasting blood glucose (FBG) ≥ 126 mg/dL, glycosylated hemoglobin A1c (HbA1c) ≥ 6.5% (National Glycohemoglobin Standardization Program), or the use of anti-diabetic medication. Dyslipidemia (DL) was defined as high-density lipoprotein cholesterol < 40 mg/dL, low-density lipoprotein cholesterol ≥ 140 mg/dL, triglyceride ≥ 150 mg/dL, or the use of lipid-lowering medication. Serum creatinine was measured enzymatically, whereas estimated glomerular filtration rate (eGFR) was calculated using the Modification of Diet in Renal Disease equation with the Japanese coefficient^46^. Previous cardiovascular disease and previous cerebrovascular disease were determined by self-questionnaire.

### Definition of HUA

Hyperuricemia was defined as serum uric acid > 7.0 mg/dL according to the Japanese guidelines for the management of hyperuricemia and gout (version 3).

### Measurements

FBG, HbA1c, high-density lipoprotein cholesterol, low-density lipoprotein cholesterol, and triglyceride levels were measured. All blood analyses were performed at a local laboratory. The analytical methods were not standardized across laboratories; however, they were based on the Japan Society of Clinical Chemistry-recommended methods for laboratory tests, which have been widely accepted by laboratories throughout Japan.

### Endpoint and follow-up

After obtaining permission from the Ministry of Health, Labour and Welfare, we accessed the database containing the certificates for all deaths that occurred between 2008 and 2015. All subjects were prospectively followed for a median 1,371 days (interquartile range 959–1,820 days). The endpoint was AD-related death, including deaths due to aortic dissection and aortic aneurysmal rupture. The cause of death was determined by reviewing the death certificates and classified based on the death code (International Classification of Diseases, 10th revision). Death from rupture of aortic aneurysm was defined as the death code [I71.1], [I71.3], and [I71.8]. Death from aortic dissection was defied as the death code [I71.0].

### Statistical analysis

The sample size was calculated based on the sample size formula for the proportional-hazard regression model according to a previous report^47^. The normality of continuous variables was checked using a Kolmogorov–Smirnov–Lilliefors test. Continuous and categorical variables were compared using t-tests and chi-square tests, respectively. Survival curves were constructed using the Kaplan–Meier method and compared using log-rank tests. Significant predictors (P < 0.05) in the univariate Cox proportional hazard regression analysis were screened for using a Bayesian method. The selected predictors were entered into a multivariate analysis. Multicollinearity was checked using the variance inflation factor. Receiver operating characteristic (ROC) curves for AD-related death were constructed and used to estimate the predictive accuracy of hyperuricemia for AD-related death. We calculated the net reclassification index (NRI) to measure the quality of improvement for the correct reclassification by the addition of hyperuricemia to the multivariate model. Values of P < 0.05 were considered statistically significant. All statistical analyses were performed using standard statistical packages (JMP version 12 [SAS Institute Inc., Cary, NC, USA] and R 3.0.2 with additional packages including Rcmdr, Epi, pROC, and PredictABEL).

### Ethical approval

All procedures of studies involving human participants were performed in accordance with the ethical standards of the institutional and/or national research committee at which the studies were conducted (Yamagata University, 2008, no. 103) and in compliance with the 1964 Helsinki Declaration and its later amendments or comparable ethical standards.

### Informed consent

This study was performed according to the Ethical Guidelines for Medical and Health Research Involving Human Subjects enacted by the Ministry of Health, Labour and Welfare of Japan (https://www.mhlw.go.jp/file/06-Seisakujouhou-10600000-Daijinkanboukouseikagakuka/0000069410.pdf; https://www.mhlw.go.jp/file/06-Seisakujouhou-10600000-Daijinkanboukouseikagakuka/0000080278.pdf). In the context of the guidelines, the investigators were not necessarily required to obtain informed consent. Nevertheless, we publicized information concerning this study on the web (https://www.fmu.ac.jp/univ/sangaku/data/koukai_2/2771.pdf) and ensured opportunities for the research subjects to refuse the use of their personal information.

## Supplementary information

Supplementary information.

## Data Availability

Data cannot be shared publicly due to ethical restrictions on sharing data publicly. The protocol of this project (Research on the Positioning of Chronic Kidney Disease in Specific Health Check and Guidance in Japan) determined that analytical data were distributed only to the members of steering committee to avoid any possibility that someone else identify individuals of this cohort. Because the data contain potentially identifying information (i.e. prefectural number and date of health checking), our institutional ethics committee has imposed them. Also, data had been obtained with the protocol approved by the relevant institutional ethical review board. Data are available upon request, please contact Department of Chronic Kidney Disease Initiatives; Fukushima Medical University School of Medicine; 1-Hikarigaoka, Fukushima 960-1295, Japan; Phone & Fax: + 81-24-547-1898; E-mail dckdi@fmu.ac.jp.
