# Supplementary material for: Impact of hyperuricemia on mortality related to aortic diseases: a 3.8-year nationwide community-based cohort study
Source: Sci Rep. 2020 Aug 31;10:14281. doi: 10.1038/s41598-020-71301-6 (PMC7459289; doi:10.1038/s41598-020-71301-6)

Impact of Hyperuricemia on Mortality Related to Aortic Diseases: A 3.8-year Nationwide Community-based Cohort Study

Yoichiro Otaki[1], Tetsu Watanabe[1], Tsuneo Konta[1], Masafumi Watanabe[1], Koichi Asahi[2], Kunihiro Yamagata[2], Shouichi Fujimoto[2], Kazuhiko Tsuruya[2], Ichiei Narita[2], Masato Kasahara[2], Yugo Shibagaki[2], Kunitoshi Iseki[2], Toshiki Moriyama[2], Masahide Kondo[2], and Tsuyoshi Watanabe[2]

[1]Department of Cardiology, Pulmonology, and Nephrology, Yamagata University School of Medicine, Yamagata, Japan

[2]Steering Committee of Research on Design of the Comprehensive Health Care System for Chronic Kidney Disease (CKD) Based on the Individual Risk Assessment by Specific Health Check, Fukushima, Japan

Correspondence: Tetsu Watanabe, MD, PhD, Department of Cardiology, Pulmonology, and Nephrology, Yamagata University School of Medicine, 2-2-2 Iida-Nishi, Yamagata, Japan 990-9585; E-mail: tewatana@med.id.yamagata-u.ac.jp; Phone: +81-23-628-5302; Fax: +81-23-628-5305

Supplementary results

Hyperuricemia and AD-related death in men

To examine the sex difference in the impact of hyperuricemia on the AD-related death, we performed sub-analysis by gender. Kaplan-Meier analysis demonstrated that subjects with hyperuricemia had a higher rate of AD-related death than those without hyperuricemia in men (Supplementary figure 1A). Incident AD-related deaths significantly increased with increasing uric acid level (Supplementary figure 2). To determine the risk factors for predicting AD-related deaths in men, we performed univariate and multivariate Cox proportional hazard regression analyses. In the univariate analysis, uric acid was significantly associated with AD-related mortality (Supplementary table), while age, sex, HT, smoking, previous cardiovascular disease, previous cerebrovascular disease, and eGFR were also related to AD-related mortality. Multivariate Cox proportional hazard regression analysis demonstrated that hyperuricemia was an independent predictor of future AD-related deaths after the adjustment for age, sex, HT, smoking, previous cardiovascular disease, previous cerebrovascular disease, and eGFR (hazard ratio, 1.189; 95% confidence interval, 1.003–1.412; P = 0.0468; Table 2).

Uric acid level and AD-related death in women.

There were 2.0% and 0.5% of women whose uric acid (UA) level greater than 7.0 and 8.0 mg/dL, respectively. Therefore, women were divided into two groups based on the serum UA level: high UA group, UA > 5.0 mg/dL, n = 92,115; and low UA group, UA ≤ 5.0 mg/dL. Kaplan-Meier analysis demonstrated that high UA group had a higher rate of AD-related death than low UA group in women

(Supplementary figure 1B). Although incident AD-related deaths were higher in high UA group than in low UA group, there were no significant difference in incident AD-related deaths among groups stratified by 1 mg/dL increments of UA (Supplementary figure 2). The impact of hyperuricemia on AD-related deaths in women could not be investigated in the multivariate Cox proportional hazard regression analysis in women due to lack of statistical power.

Supplementary table. Univariate and multivariate Cox proportional hazard analyses of predicting AD-related death in men.

Variables	Hazard Ratio	95% confidence interval	P value
Univariate analysis			
Age, per-1year increase	1.085	1.044-1.128	<0.0001
Hypertension	9.366	3.879-30.777	<0.0001
Smoking	1.598	0.980-2.557	0.0540
Previous cardiovascular disease	2.262	1.091-4.209	0.0300
Previous cerebrovascular disease	2.667	1.179-5.233	0.0210
eGFR, per-1SD increase	0.526	0.406-0.681	<0.0001
Uric acid, per-1SD increase	1.295	1.105-1.516	0.0013
Multivariate analysis			
Age, per-1year increase	1.068	1.024-1.113	0.0022
Hypertension	7.000	2.877-23.101	<0.0001
Smoking	2.473	1.501-3.998	0.0005
Previous cardiovascular disease	1.502	0.716-2.839	0.2630
Previous cerebrovascular disease	1.690	0.739-3.368	0.1966
eGFR, per-1SD increase	0.666	0.508-1.501	0.0033
Uric acid, per-1SD increase	1.189	1.003-1.412	0.0468

AD, aortic artery disease; eGFR, estimated glomerular filtration rate.

Supplementary figure legends.

Supplementary figure 1. Kaplan-Meier analysis of aortic artery disease (AD)–related deaths in subjects with versus those without hyperuricemia in men (A). Kaplan-Meier analysis of AD–related deaths between high uric acid (UA) group (UA > 5.0 mg/dL) and low UA group (UA ≤ 5.0 mg/dL) in women.

Supplementary figure 2. Association of aortic disease (AD)–related death per 100,000 person-years with serum uric acid levels.

Supplementary Figure 1A

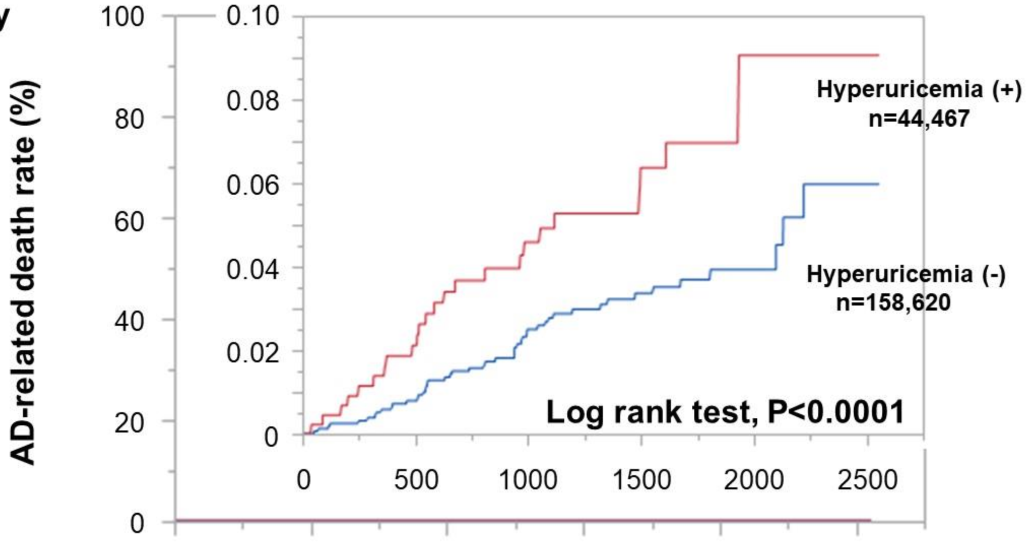

No at risk	0	500	1000	1500	2000	2500
Hyperuricemia (-)	158,620	143,386	114,597	68,561	18,935	1,061
Hyperuricemia (+)	44,467	39,712	31,259	18,181	4,003	184

Supplementary Figure 1B

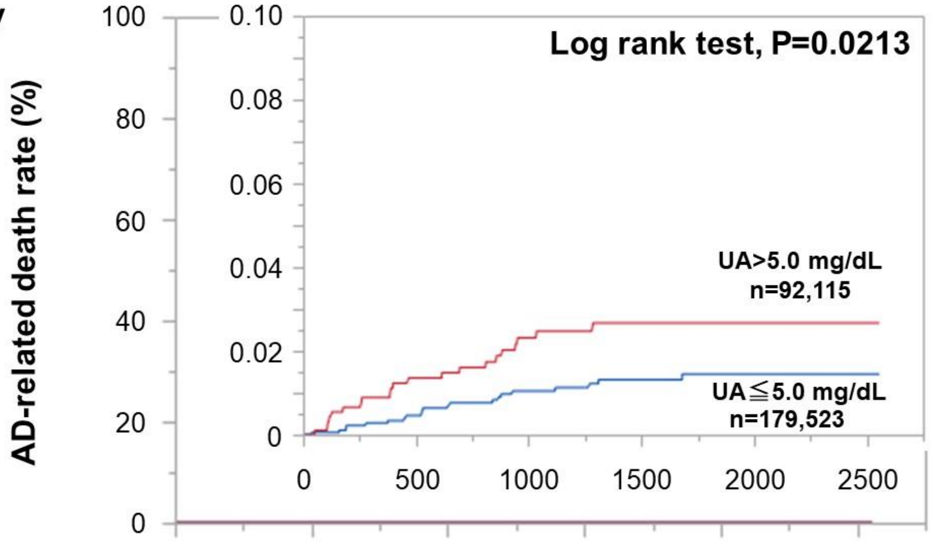

No at risk	0	500	1000	1500	2000	2500
UA>5.0 mg/dL	179,523	165,381	137,212	86,340	24,413	1,247
UA≦5.0 mg/dL	92,115	83,040	66,612	39,856	9,409	416

Supplementary figure 2

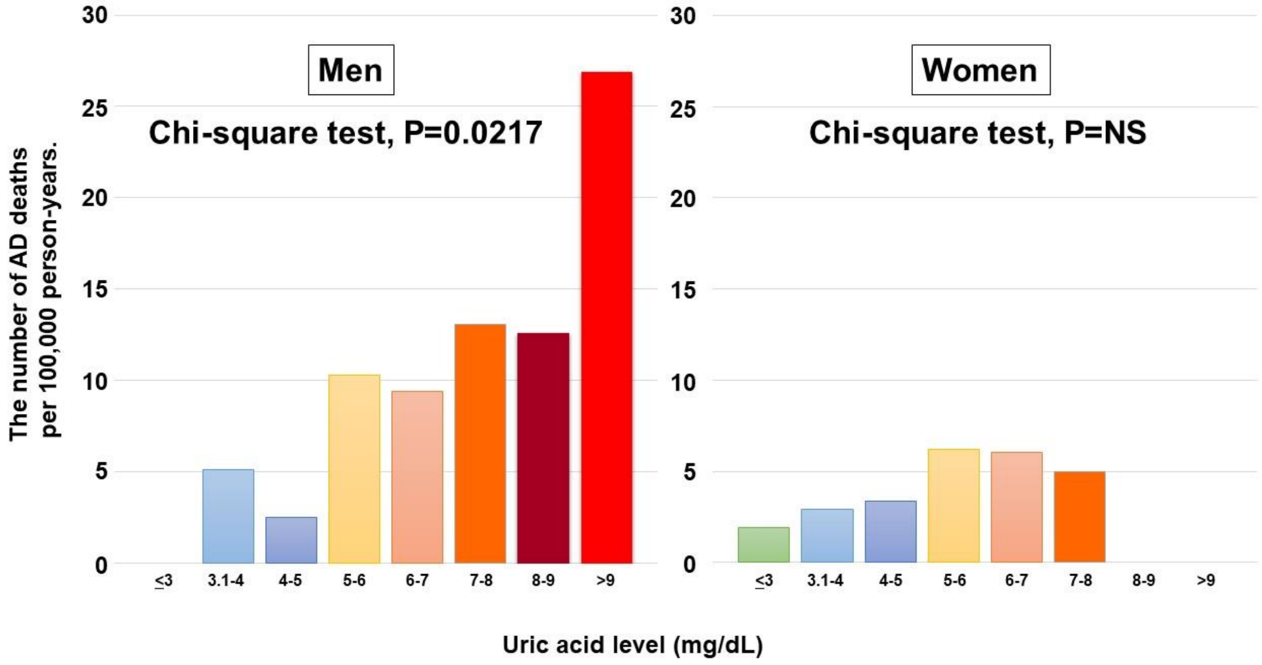

Supplement: Supplementary file 1 — Supplementary information. [file 41598_2020_71301_MOESM1_ESM.pdf]
